# Osmanicin, a Polyketide Alkaloid Isolated from *Streptomyces osmaniensis* CA-244599 Inhibits Elastase in Human Fibroblasts

**DOI:** 10.3390/molecules24122239

**Published:** 2019-06-15

**Authors:** Mamdouh N. Samy, Géraldine Le Goff, Philippe Lopes, Katerina Georgousaki, Sentiljana Gumeni, Celso Almeida, Ignacio González, Olga Genilloud, Ioannis Trougakos, Nikolas Fokialakis, Jamal Ouazzani

**Affiliations:** 1 Institut de Chimie des Substances Naturelles ICSN, Centre National de la Recherche Scientifique, Avenue de la Terrasse, 91198 Gif-sur-Yvette, France; mamdouhnabil.2006@yahoo.com (M.N.S.); Geraldine.LEGOFF@cnrs.fr (G.L.G.); Philippe.LOPES@cnrs.fr (P.L.); 2 Department of Pharmacognosy & Natural Products Chemistry, Faculty of Pharmacy, National and Kapodistrian University of Athens, 15771 Athens, Greece; kat_georgousaki@hotmail.com (K.G.); fokialakis@pharm.uoa.gr (N.F.); 3 Department of Cell Biology and Biophysics, Faculty of Biology, National and Kapodistrian University of Athens, 15784 Athens, Greece; sgumeni@biol.uoa.gr (S.G.); itrougakos@biol.uoa.gr (I.T.); 4 Fundación MEDINA, Parque Tecnológico de Ciencias de la Salud, 18016 Granada, Spain; celsoguerreiro@gmail.com (C.A.); ignacio.gonzalez@medinaandalucia.es (I.G.); olga.genilloud@medinaandalucia.es (O.G.)

**Keywords:** *Streptomyces osmaniensis*, in situ solid-phase extraction, osmanicin, alkaloids, elastase inhibition

## Abstract

The strain *Streptomyces osmaniensis* CA-244599 isolated from the Comoros islands was submitted to liquid-state fermentation coupled to in situ solid-phase extraction with amberlite XAD-16 resin. Elution of the trapped compounds on the resin beads by ethyl acetate afforded seven metabolites, osmanicin (**1**), streptazolin (**2**), streptazone C (**3**), streptazone B_1_ (**4**), streptenol C (**5**), nocardamine (**6**) and desmethylenylnocardamine (**7**). Osmanicin (**1**) is a newly reported unusual scaffold combining streptazolin (**2**) and streptazone C (**3**) through a Diels-Alder type reaction. Experimental evidence excluded the spontaneous formation of **1** from **2** and **3**. The isolated compounds were evaluated for their ability to inhibit elastase using normal human diploid fibroblasts. Compound **1** exhibited the most potent activity with an IC_50_ of 3.7 μM.

## 1. Introduction

The *Streptomyces* genus belongs to the gram-positive actinobacteria phylum and is among the most prolific producers of diverse and bioactive secondary metabolites. These metabolites belong to different structural classes, including polyketides, peptides, terpenoids, alkaloids and hydrazides [1], which exhibit a wide range of biological activities such as antibiotic (vancomycin, erythromycin and tetracycline), antifungal (amphotericin B), anticancer (mitomycin C), antiparasitic (ivermectin) and immunosuppressive (rapamycin) activities [2].

Although *Streptomyces* species from terrestrial and marine ecosystems have been intensively investigated in recent decades, there still remains a pool of unexplored diversity of secondary metabolites. This discovery pipeline is continuously fed through innovative experimental strategies [3] and genome-based approaches [4].

The present study was conducted in the frame of our screening program to discover bioactive metabolites from microorganisms of global biodiversity with skin related anti-wrinkle properties [5]. Among our targets, elastase, that is a matrix metalloproteinase (MMP), constitutes a key enzyme involved in the degradation of proteins contained in skin connective tissue. Elastases are serine proteases that break elastin fibers and regulates, together with collagenases, the mechanical properties of the skin including elasticity, strength, tissue remodeling, and wound healing capacity [6]. Under normal physiological conditions, the activity of elastase is precisely regulated to ensure skin tissue homeostasis. Following oxidative stress or UV light exposure, elastase is overexpressed, resulting in skin disorders such as premature skin aging, inflammation, or more seriously, degenerative diseases [7]. Moreover, the age-related upregulation of fibroblast elastase was correlated to an increase of advanced glycation end products [8]. The search for natural substances able to inhibit elastase is thus of great interest for the cosmetic and/or pharmaceutical industries.

During our screening program a collection of 100 selected microbial strains, covering worldwide diversity, were grown in ten different nutritional conditions and their extracts were screened as potential elastase inhibitors. Among them, an extract from the *Streptomyces* strain CA-244599 exhibited a significant inhibitory effect against elastase in cell free assays and then in a human skin fibroblast cell line (CCD25SK) while had no cytotoxicity in cancer cell lines (A2058, HepG2). Phylogenetic analysis based on 16S rRNA gene sequences has confirmed that the isolated strain belongs to the genus *Streptomyces* and is closely related to the species *Streptomyces osmaniensis* OU-63^T^. To the best of our knowledge the type species *S. osmaniensis* has never been investigated for its bioactive metabolites. 

In the frame of the current work, we describe herein the scale-up isolation and characterization of a new elastase inhibitor together with six previously described metabolites that have potential applications in the cosmetic industry.

## 2. Results and Discussion

**Figure 1:**
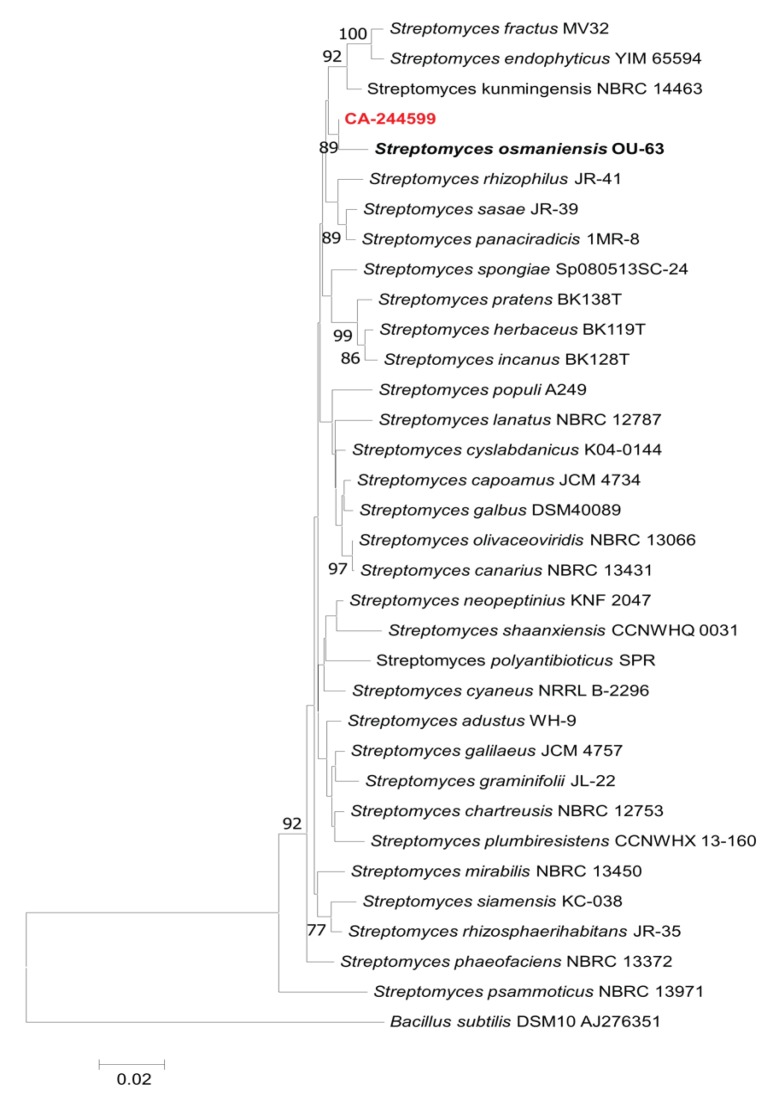
Phylogenetic analysis of Actinomycete isolate CA-244599.

The *Streptomyces* strain CA-244599 was isolated from a soil sample collected from a savannah environment at Bangouamafsakoa, in the Comoros Islands. The strain was confirmed as *Streptomyces* by molecular analysis according to its 16S rRNA and tentatively assigned to the species *S. osmaniensis* by following phylogenetic analysis (Figure 1).

**Figure 2:**
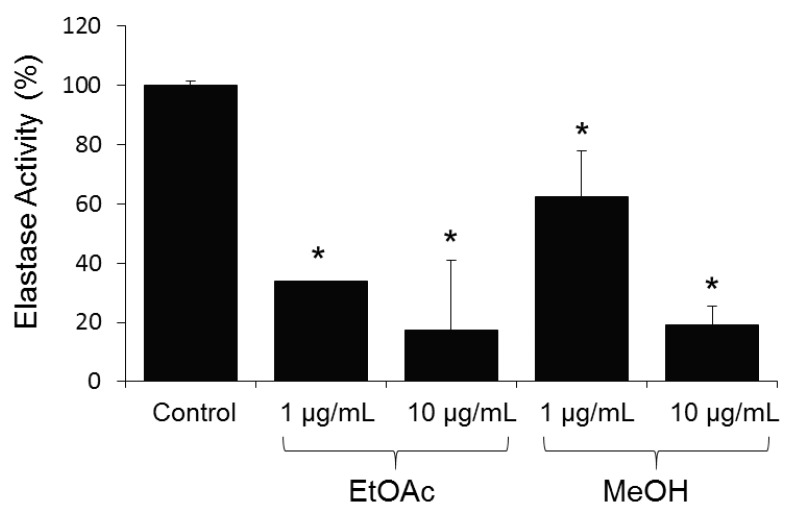
Relative (%) elastase inhibitory activity in normal human BJ fibroblasts after 24 h of treatment with shown extracts at the concentration of 1 and 10 µg/mL. Values from controls (cells treated without the extract) were set to 100%. Data are presented as mean ± SD (*n* ≥ 3). The statistical differences observed in the graphic are significant when compared to control samples, * *p* < 0.05.

The strain was cultivated in Potatoes Dextrose Broth (PDB) medium in a 20 L fermenter (12 L working volume) coupling liquid-state fermentation with in situ solid-phase extraction using Amberlite XAD-16 neutral resin [9,10]. After five days of cultivation, the resin was recovered by filtration and washed extensively with water. The compounds were eluted from the resin by ethyl acetate followed by methanol, and analyzed by HPLC coupled with PDA, LSD, MS detectors and were evaluated for their activity in cell-based (BJ cells) assays at the concentration of 1 and 10 µg/mL (Figure 2).

**Figure 3:**
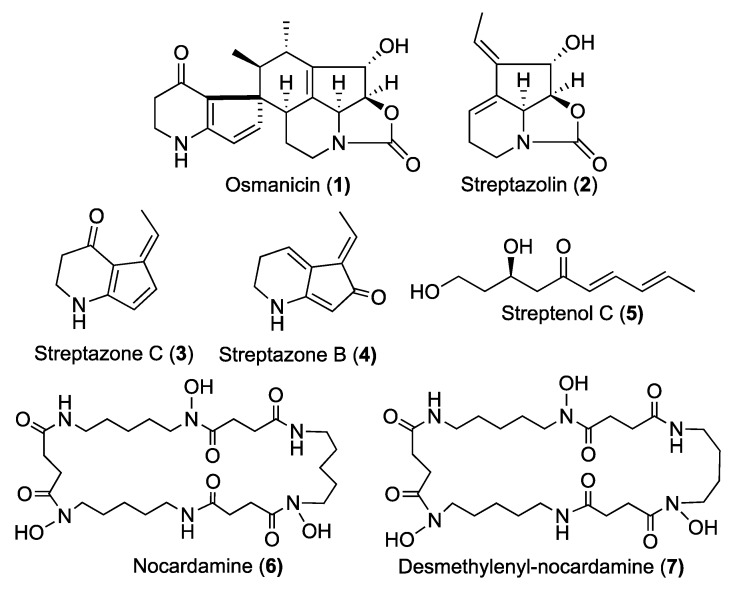
Compounds isolated from *Streptomyces osmaniensis* CA-244599.

All analyzed extracts displayed a statistically significant inhibition of elastase activity in a dose dependent way. Specifically, the ethyl acetate extract showed the highest inhibition by 66.2% at 1 µg/mL and 82.4% at 10 µg/mL. The analytical HPLC chromatogram of this extract indicated the presence of 7 peaks, which were purified by Flash chromatography followed by semi-preparative HPLC. Among them, the structure of a previously undescribed compound osmanicin (**1**) was elucidated using 1D- and 2D-NMR spectroscopic analyses and high-resolution mass spectrometry. Besides the new compound, the previously described streptazolin (**2**) [11], streptazone C (**3**) and B (**4**) [12], streptenol C (**5**) [13], nocardamine (**6**) [14] and desmethylenylnocardamine (**7**) [15] were also isolated (Figure 3).

The molecular formula of **1** was deduced as C_21_H_24_N_2_O_4_ on the basis of HRESIMS with *m/z* 369.1826 [M + H]^+^. The IR spectrum showed absorptions indicating the presence of hydroxyl groups (3279 cm^−1^) and a carbonyl group (1737 cm^−1^). The ^1^H-NMR spectrum of **1** (Table 1) displayed signals corresponding to two doublet olefinic protons at δ_H_ 6.33 (d, *J* = 5.8 Hz) and 6.78 (d, *J* = 5.8 Hz), two doublet methyls at δ_H_ 0.62 (d, *J* = 7.1 Hz) and 1.05 (d, *J* = 7.4 Hz), three oxymethine protons at δ_H_ 4.53 (br s), 4.68 (m) and 4.69 (m), as well as three methine and four methylene protons (refer to Supplementary Information). 

**Figure 4:**
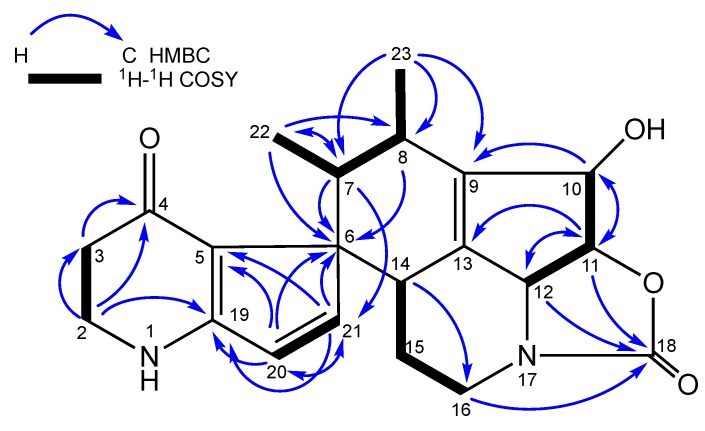
Key COSY and HMBC correlations for compound **1**.

The ^13^C-NMR spectrum of **1** (Table 1) revealed the presence of 21 carbon signals, including two carbonyls at δ_C_ 185.7 and 160.0, two methyls at δ_C_ 12.8 and 13.4, in addition to two olefinic carbons, three oxymethines, three methines, four methylenes and four quaternary carbon signals. Comparing spectroscopic data with those from literature, we found that compound **1** consisted of the attachment of streptazolin (**2**) and streptazone C (**3**). The attachment of the two compounds was confirmed by ^1^H−^1^H COSY and HMBC correlations (Figure 4). The ^1^H−^1^H COSY correlations confirmed the linkage of H_3_-22/H-7/H-8/H_3_-23. The methyl protons H_3_-22 (δ_H_ 1.05) showed significant HMBC correlations with C-6 (δ_C_ 61.0), C-7 (δ_C_ 38.9) and C-8 (δ_C_ 33.2), confirming its attachment to C-6, whereas the other methyl group H_3_-23 (δ_H_ 0.62) was correlated with C-7 (δ_C_ 38.9), C-8 (δ_C_ 33.2) and C-9 (δ_C_ 141.3), indicating its attachment to C-9. This was also supported by key HMBC correlations between the methine proton H-7 (δ_H_ 2.81) and C-6 (δ_C_ 61.0), C-21 (δ_C_ 154.6) and C-22 (δ_C_ 12.8) and between the methine proton H-8 (δ_C_ 2.60) and C-6 (δ_C_ 61.0) and C-23 (δ_C_ 13.4). These findings were strongly indicative to the cyclization between the two exocyclic ethylidene side chains. 

The spectroscopic data of the isolated streptazolin are identical to previous publications reporting the absolute stereochemistry of **2** [16,17]. We thus attributed the configuration of C-10, C-11 and C-12 in **1**.

ROE correlations between H-11 and H-12 and the absence of correlation between H-10, H-12, and H-11, H-12 confirmed the stereochemistry of the streptazolin moiety.

**Figure 5:**
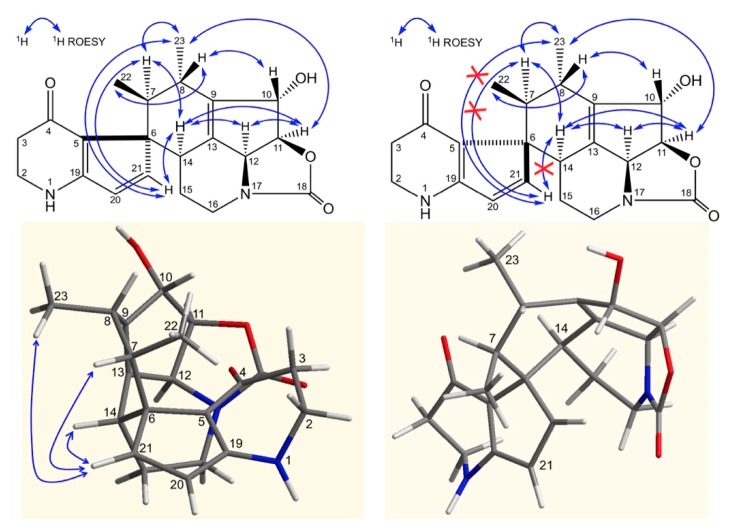
Confirmation of the stereochemistry at C-6.

In Figure 5, ROE correlations between H-11, H-12 and H-14, between H-14 and H-7 and between H10 and H8, allow the assignment of the stereochemistry at the newly generated asymmetric centers C-14, C-7 and C-8. The configuration of the quaternary carbon 6 was deduced from ROE correlations between H-21 and H-14, H-22, H-23 and confirmed according to the 3D structure obtained by Chem3D (ChemBio3D, Perkin Elmer) (Figure 5) (ROE, Rotating-frame Overhauser Enhancement).

Therefore, compound **1** exhibits a *6S, 7S, 8S, 10S, 11S, 14S* configuration and was named osmanicin.

**Figure 6:**
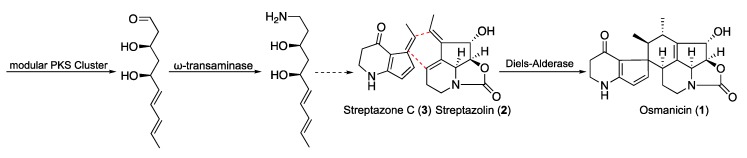
The proposed biosynthetic pathway of osmanicin (**1**).

The complete biosynthesis of polyketide alkaloids in *Streptomyces* is not yet elucidated. However, some common features were recently published involving a type I polyketide synthase (PKS) associated with a ω-transaminase located genetically close to the PKS cluster [18]. This pathway is considered common to all the polyketide alkaloids especially streptazones and streplazolin. Based on these considerations, compound **1** may derives from a combination between streptazone C and streplazolin through a Diels-Alder type reaction (Figure 6) [19,20]. Although Diels-Alder cycloaddition is a key reaction in organic chemistry, its enzymatic counterpart has been highlighted in different biosynthesis of natural products [20]. Even if most of the reactions are intramolecular, few intermolecular cases were reported, even if they have been the subject of particular controversy [21,22]. Recently, many groups have engaged the optimization of specific Diels-Alder biocatalysts by combining targeted mutagenesis, computational refinement and directed evolution [23,24]. We recently reported the case of sporochartine involving a tentative Diels-Alderase intermolecular cycloaddition [25]. Sporochartine and osmanicin represents the targets of our future investigations in the field.

Streptazolin (**2**), streptazone C (**3**), streptazone B (**4**), nocardamine (**6**) and desmethylenyl-nocardamine (**7**) were previously reported from terrestrial *Streptomyces* spp as previously indicated.

**Figure 7:**
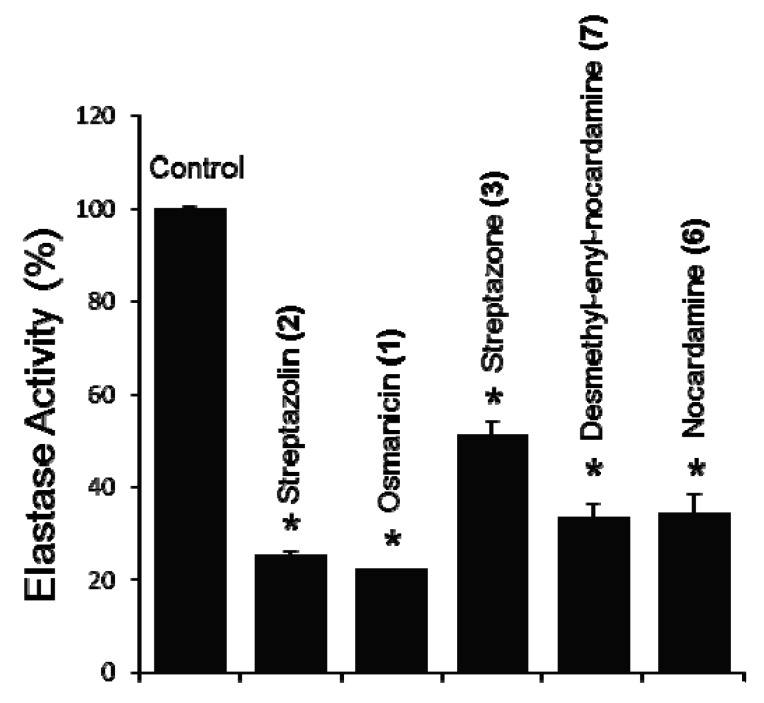
Relative (%) elastase inhibitory activity in human fibroblasts after 24 h of treatment with pure compounds at the concentration 5 μM. Values from controls (cells treated without the extract) were set to 100%. Data are presented as mean ± SD (*n* = 3). Shown differences are significant vs. control samples, * *p* < 0.05.

The compounds were then screened on a cell-based bioassay for their effect on elastase activity. Normal human diploid fibroblasts (BJ cells) were incubated with 5 μM of concentration for each compound, and after 24 h of incubation elastase activity was measured. Compounds **1**, **2**, **3**, **6** and **7** exhibit a statistically significant inhibition ranging from 48.8 to 77.7% compared to the non-treated cells, with the highest inhibition being observed for osmanicin (**1**) (Figure 7). Compounds **4** and **5** did not exhibit any significant activity (not shown).

**Figure 8:**
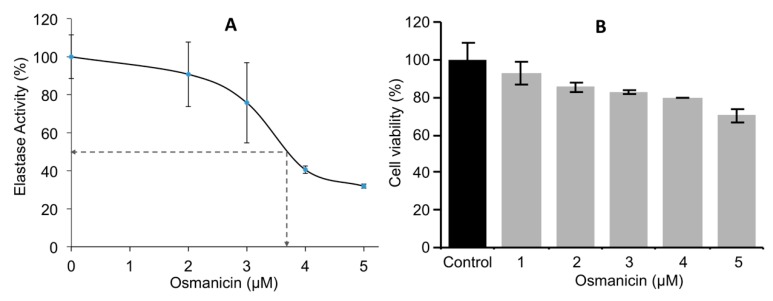
Determination of the IC_50_ inhibitory concentration of osmanicin against elastase (**A**) and cell survival after osmanicin treatment (**B**). (**A**) The half maximal inhibitory concentration (IC_50_) of osmanicin against elastase was calculated by plotting the graph between the different concentrations used (1 μM to 5 μM) and the % inhibition of elastase activity. Data are presented as mean ± SD (*n* ≥ 3). (**B**) Relative (%) survival (MTT assay) of BJ fibroblasts exposed to the indicated concentrations of osmanicin for 24 h. Data are presented as mean ± SD (*n* ≥ 3).

Normal fibroblasts were then incubated for 24 h at different concentrations to obtain the IC_50_ inhibitory values of osmanicin against elastase. The IC_50_ value of elastase inhibition was found to be 3.7 μM (Figure 8A). Furthermore, BJ normal human fibroblasts were treated for 24 h with increasing concentrations of osmanicin (1 μM to 5 μM) in order to evaluate the toxicity of the compound. Under these experimental conditions, osmanicin showed some minor toxicity (*p* = 0.0425) only at the concentration of 5 μM (Figure 8B).

Given the overall lifespan increase in the western world, there is a growing interest in skin health and in products that likely delay skin aging processes. The main symptoms of skin aging are the wrinkling and sagging due to the loss of flexibility and elasticity. These processes are (among others) driven by the enzymatic activity of elastases. Reportedly, aging leads to the upregulation of elastase activity and therefore to tissue destruction and loss of elasticity [26]. We show herein that the ethyl acetate extract of the strain *Streptomyces osmaniens* is CA-244599 exerts an inhibitory activity against elastase in normal human fibroblasts. Our assays were performed in human fibroblasts elastase, in order to examine the proprieties of the isolated extracts/pure compounds in preventing the proteolytic degradation of dermal elastic fibers. Our data showed a significant decrease of elastase activity in a dose-dependent manner. Therefore, the herein described extracts/pure compounds from *Streptomyces osmaniensis* can be likely considered as anti-wrinkling agents in the industry of cosmetics. Furthermore, we fully characterized the extract of *Streptomyces osmaniensis*, and our data revealed that all the isolated compounds inhibited the activity of skin fibroblasts elastase. In addition, we identified osmanicin a new, non-previously described metabolite, which showed the greatest inhibitory (77%) effect on elastase activity. Overall, our data suggest that extracts of *Streptomyces osmaniensis*, along with osmanicin may be used as an effective biomaterial in skin anti-aging treatments as it can likely reduce the degradation of the dermal fibers by inhibiting elastase activity. 

## 3. Materials and Methods

### 3.1. General Experimental Procedures

Optical rotations [α]_D_ were measured using an Anton Paar MCP-300 polarimeter at 589 nm. The IR spectra were obtained using a Perkin-Elmer Spectrum 100 model instrument. NMR experiments were performed using a Bruker Avance 500 MHz spectrometer (Bruker Biospin, Wissembourg, France). All the spectra were acquired in CD_3_OD (δ_H_ 3.31 ppm and δ_C_ 49.15 ppm) at room temperature. High-resolution mass spectra were obtained on a Waters LCT Premier XE spectrometer equipped with an ESI-TOF (electrospray-time of flight) by direct infusion of the purified compounds. Pre-packed silica gel Redisep columns were used for flash chromatography using a Combiflash-Companion chromatogram (Serlabo, Entraigues sur La Sorgue, France). All other chemicals and solvents were purchased from SDS (eypin, France). 

Analytical HPLC system consisted of an Alliance Waters 2695 controller coupled with a PhotoDiode Array Waters 2996, an evaporative light-scattering detector ELSD Waters 2424 detector and a mass detector Waters QDa. Sunfire C_18_ column (4.6 × 150 mm, 3.5 μm) was used with a flow rate of 0.7 mL/min. The elution gradient consisted of a linear gradient from 100% solvent A to 100% solvent B in 40 min, then 10 min at 100% B (Solvent A: H_2_O + 0.1 HCOOH, Solvent B: ACN + 0.1% HCOOH). Preparative HPLC was performed on a semi-preparative Sunfire C_18_ column (10 × 250 mm, 5 μm) using a Waters autosampler 717, a pump 600, a photodiode array detector 2996 and an ELSD detector 2420 (Waters, Guyancourt, France). 

### 3.2. Bacterial Strain

The *Streptomyces* strain CA-244599 was isolated from a dry soil sample collected in the savannah of Bangouamafsakoa in the Comoros Islands. This soil was air dried and suspended in sterile water. The soil suspension was serially diluted, plated on selective isolation media and incubated at 28 °C for at least six weeks. The strain was isolated from an NZ-amine-based agar medium containing nalidixic acid (20 µg/mL). The colony was purified on yeast-malt extract glucose medium (ISP2) and preserved as frozen agar plugs in 10% glycerol.

### 3.3. 16. S rDNA Sequence and Phylogenetic Analysis

Total genomic DNA was recovered and purified as previously described [27] from the strain grown in ATCC-2 liquid medium (0.5% yeast extract (Difco, Franklin Lakes, NJ, USA), 0.3% beef extract (Difco), 0.5% peptone (Difco), 0.1% dextrose (Difco), 0.2% starch from potato (Panreac, Barcelona, Spain), 0.1% CaCO3 (E. Merck, Darmstadt, Germany) and 0.5% NZ amine E (Sigma, St Louis, MO, USA). DNA preparations were used as template DNA for Taq Polymerase. PCR primers fD1 and rP2 were used for amplifying the 16S ribosomal RNA gene of the strain [28,29]. Reactions were performed in a final volume of 50 µl containing 0.4 µM of each primer, 0.2 mM of each of the four deoxyribonucleotide triphosphates (Roche, Indianapolis, IN, USA), 5 µl of extracted DNA, 1U Taq polymerase (Appligene, Watford, UK) with its recommended reaction buffer. PCR amplifications were performed in a Peltier Thermal Cycler PTC-200, according to the following profile: 5 min at 95 °C and 40 cycles of 30 s at 94 °C, 30 s at 52 °C for and 1 min at 72 °C, followed by 10 min at 72 °C. The amplification products were analyzed by electrophoresis in 2% (*w*/*v*) pre-cast agarose gels stained with ethidium bromide (E-gel 2%, 48 wells, Invitrogen, Carlsbad, CA, USA). PCR products were sent to Secugen (Madrid, Spain) for sequencing and were purified and used as a template in sequencing reactions using the primers fD1 and rP2 [23,24]. Amplified DNA fragments were sequenced using the ABI PRISMDYE Terminator Cycle sequencing kit and fragments were resolved using the ABI3130 genetic analyzer (Applied Biosystems, Foster City, CA, USA). Partial sequences were assembled and edited using the Assembler contig editor component of Bionumerics (ver 6.6) analysis software (Applied Maths NV, Sint-Martens-Latem, Belgium).

The almost-complete 16S rRNA gene sequence (1,329 nucleotides Genbank access MH443355) of strain CA-244599 was compared to those deposited in public databases and the EzBiocloud server (https://www.ezbiocloud.net) [30]. The strain exhibited the highest similarity (99.10%) with was *Streptomyces osmaniensis* OU-63^T^ (FJ613126), using EzBiocloud and GenBank sequence similarity searches and homology analysis. Phylogenetic and molecular evolutionary analyses were conducted using MEGA version 6 [31]. Multiple alignment was carried out using CLUSTALX [32], integrated in the software. The phylogenetic analysis, based on the Neighbor-Joining method [33] using matrix pairwise comparisons of sequences corrected with Jukes and Cantor algorithm [34], shows that the strain is closely related to the type strain *Streptomyces osmaniensis* OU-63^T^ and this relatedness is well supported in the analysis by the bootstrap value (97%). The morphological, 16S rRNA gene sequence and phylogenetic data were indicative that strain CA-244599 is a member of the genus *Streptomyces* and the strain is tentatively referred as *Streptomyces osmaniensis* CA-244599. 

### 3.4. Fermentation, Isolation and Structure Elucidation of Compounds

The original production was performed in 1L volume of the liquid medium FRM. The first seed culture of the strain CA-244599 was prepared by inoculating 50 mL of seed medium (soluble starch (20 g/L), dextrose (10 g/L), NZ amine EKC (Sigma) (5 g/L), Difco beef extract (3 g/L), Bacto peptone (5 g/L), yeast extract (5 g/L), and CaCO3 (1 g/L), adjusted to pH 7.0 with NaOH before addition of CaCO_3_), in 250 mL flasks with 2.5 mL of a frozen inoculum stock of the producing strain and incubating the tube at 28 °C and 220 rpm agitation for 48 h. A 5% aliquot of the seed culture was transferred to eight 500 mL flasks containing 150 mL of the FRM production medium (glycerol (Panreac 141339) (20 g/L), Dextrin from corn Type I (SIGMA D2006) (20 g/L), Bacto Soytone (Difco 243620) (3 g/L), (NH_4_)_2_SO_4_ (Panreac 141140) (2 g/L) and CaCO3 (Merck 8605747) (3 g/L), adjusted to pH 7.4 prior to the addition of CaCO_3_). The flasks were incubated at 28 °C for seven days in a rotary shaker at 220 r.p.m. and 70% humidity before been harvested.

Batch fermentation of *Streptomyces osmaniensis* CA-244599 was conducted in a 15 L fermentor (Chemap 20 L unit) in a PDB medium over five days at 28 °C with an aeration rate of 0.5 volumes of air per volume per minute, 100 rpm agitation and pH of 6.6. Amberlite XAD-16 (30 g/L) was added prior to sterilization to allow the in situ trapping of the microbial metabolites.

The XAD-16 resin was separated from the broth culture via filtration and washed with water before being eluted successively with EtOAc and MeOH (3 × 500 mL, each). The eluate was concentrated to dryness in vacuo yielding 1.26 g of EtOAc and 8.59 g of MeOH extracts.

The EtOAc extract (1.26 g) was subjected to flash chromatography on a Combiflash Companion using a Redisep 40 g silica column, with a heptane−ethyl acetate mixture serving as the eluent giving 10 fractions. The fourth fraction (132.5 mg) was purified by HPLC (30% CH_3_CN with 0.1% formic acid) to afford compound **3** (67.4 mg). The sixth fraction (50.6 mg) was purified by HPLC (30% CH_3_CN with 0.1% formic acid) to furnish compound **4** (11.0 mg). The seventh fraction (62.3 mg) was purified by HPLC (H_2_O−CH_3_CN gradient supplemented with 0.1% formic acid (100−0 to 0−100) to produce compound **5** (4.2 mg). The tenth fraction (190.4 mg) was purified by HPLC (H_2_O−CH_3_CN gradient supplemented with 0.1% formic acid (100−0 to 0−100) to give compounds **1** (8.9 mg) and **2** (2.4 mg).

The MeOH extract (4.25 g) was subjected to flash chromatography on a Combiflash Companion using a Redisep 80 g silica column, with a CH_2_Cl_2_−MeOH mixture serving as the eluent giving 9 fractions. The seventh fraction (315.4 mg) was purified by HPLC (H_2_O−CH_3_CN gradient supplemented with 0.1% formic acid (100−0 to 0−100)) to give compounds **6** (2.3 mg) and **7** (1.7 mg).

Osmanicin (**1**): Yellowish residue; [α]^25^_D_ ‒92 (c 0.10, MeOH); UV (MeOH) λmax (log ε) 243 (5.79), 345 (5.44) nm; IR (film) ν_max_ 3279, 2965, 1737, 1616, 1571, 1544, 1519, 1451, 1412, 1329, 1218, 1098, 1032, 767 cm^−1^; ^1^H-NMR (300 MHz, CD_3_OD) and ^13^C-NMR (75 MHz, CD_3_OD) see Table 1; HRESIMS *m*/*z* [M + H]^+^ 369.1826 (Calcd for C_21_H_25_N_2_O_4_, 369.1814).

### 3.5. Cell Based Elastase Bioassay

Proliferating normal human diploid fibroblasts (BJ cells) were cultured as described before [35]. In order to evaluate elastase activity, the amount of released *p*-nitroaniline, which was hydrolyzed from the substrate (*N*-succinyl-Ala-Ala-Ala-*p*-nitroanilide) by elastase, was determined by measuring the absorbance at 405 nm. Thus, BJ fibroblasts were seeded into 96-well microplates and the next day they were treated with compounds **1** to **7** at a final concentration of 5 μM for each compound and of 1 μg/mL or 10 μg/mL of concentration for each extract, for 24 h. Afterwards, cells were lysed in 100 mM Tris-HCl (pH 7.6) with 0.1% Triton X-100 buffer and subsequently, 2 mM N-succinyl-Ala-Ala-Ala-p-nitroanilide (Sigma-Aldrich) was added to each well followed by incubation at 37 °C for 1 h. The absorbance at 405 nm was measured using a microplate reader Infinite 200 PRO series (Tecan Trading AG, Switzerland).

### 3.6. Determination of Osmanicin IC_50_ Inhibitory Concentration against Elastase

The inhibitory IC_50_ concentration of Osmanicin against elastase was assayed by using serial dilutions of Osmanicin (1 μM to 5 μM). After 24h of treatment, the elastase activity was measured as described above. The relative (%) inhibition per concentration of the compound was calculated and the IC_50_ value was determined; the elastase activity of control cells (treated with the solvent) was arbitrarily set to 100%.

### 3.7. Cell Survival Assay

The effect of osmanicin on the viability of BJ fibroblasts was examined using the MTT assay [36]. Briefly, cells were plated in flat-bottomed 96-well microplates and the next day they were incubated with different concentrations of osmanicin for 24 h. Following treatment, the medium was replaced by 3-(4,5-dimethylthiazol-2-yl)-2,5-diphenyltetrazolium bromide (MTT, Sigma-Aldrich) dissolved at a final concentration of 1 mg/mL in serum-free, phenol red-free medium. The formed formazan crystals were then dissolved by isopropanol and the absorbance of the solution was measured at 570 nm wavelength. Values from control cells were arbitrarily set to 100%.

### 3.8. Statistical Analysis

Experiments were performed at least in duplicate unless otherwise indicated in figure legends. For statistical analyses, MS Excel and the Statistical Package for Social Sciences (IBM SPSS; version 19.0 for Windows) were used. Statistical significance was evaluated using one-way analysis of variance (ANOVA). Data points correspond to the mean of the independent experiments and error bars denote standard deviation (SD).

## Figures and Tables

**Table 1:** NMR spectroscopic data (CD_3_OD) for osmanicin (**1**).

Osmanicin (1) ^a^			
No.	δ_C_, type	δ_H_, mult. (*J* in Hz)	HMBC
2	43.4, CH_2_	3.62, bt (7.4)	C-3, 4, 19
3	36.2, CH_2_	2.39, (dt (8.4, 3.9)	C-2, 4, 5
4	185.7, C		
5	112.4, C		
6	61.0, C		
7	38.9, CH	2.81, b quint (7.6)	C-6, 21, 22
8	33.2, CH	2.60, m	C-6, 23
9	141.3, C		
10	80.0, CH	4.68, m	C-9, 11
11	83.5, CH	4.69, m	C-10, 12, 13, 18
12	64.5, CH	4.53, b d (1.4)	C-18
13	138.7, C		
14	38.8, CH	3.36, m	C-13, 16
15	28.6, CH_2_	1.02, m 1.41, d quint (13.3, 2.9)	
16	43.6, CH_2_	3.11, td (13.3, 3.2)	C-18
		3.58, dd (5.1, 2.3)	
18	160.0, C		
19	169.2, C		
20	127.4, CH	6.33, d (5.8)	C-5, 6, 19, 21
21	154.6, CH	6.78, d (5.8)	C-5, 6, 20
22	12.8, CH_3_	1.05, d (7.4)	C-6, 7, 8
23	13.4, CH_3_	0.62, d (7.1)	C-7, 8, 9

^a 1^H and ^13^C chemical shifts were recorded at 300 and 75 MHz respectively.

## Supplementary Materials

The following are available online at https://www.mdpi.com/1420-3049/24/12/2239/s1, reporting different spectral analysis of compound **1**. Figure S1. ^1^H-NMR spectrum, Figure S2. ^13^C-NMR spectrum, Figure S3. HSQC spectrum, Figure S4. HMBC spectrum, Figure S5. ^1^H-^1^H COSY spectrum, Figure S6. ^1^H-^1^H ROESY spectrum, Figure S7. HRESIMS, Figure S8. IR spectrum.
