# Supplementary material for: Osmanicin, a Polyketide Alkaloid Isolated from Streptomyces osmaniensis CA-244599 Inhibits Elastase in Human Fibroblasts
Source: Molecules. 2019 Jun 15;24(12):2239. doi: 10.3390/molecules24122239 (PMC6630352; doi:10.3390/molecules24122239)
Supplement: Supplementary file 1 [file molecules-24-02239-s001.pdf]

SUPPORTING INFORMATION

Osmanicin, a Polyketide Alkaloid Isolated from *Streptomyces osmaniensis* CA-244599 inhibits Elastase in Human Fibroblasts

Mamdouh N. Samy[1], Géraldine Le Goff[1], Philippe Lopes[1], Katerina Georgousaki[2], Sentiljana Gumeni[3], Celso Almeida[4], Ignacio González[4], Olga Genilloud[4], Ioannis Trougakos[3], Nikolas Fokialakis[2] and Jamal Ouazzani [1,*]

[1] Institut de Chimie des Substances Naturelles ICSN, Centre National de la Recherche Scientifique, Avenue de la Terrasse 91198, Gif-sur-Yvette, France.
[2] Department of Pharmacognosy & Natural Products Chemistry, Faculty of Pharmacy, National and Kapodistrian University of Athens, Athens 15771 Greece.
[3] Department of Cell Biology and Biophysics, Faculty of Biology, National and Kapodistrian University of Athens, Athens, Greece
[4] Fundación MEDINA, Parque Tecnológico de Ciencias de la Salud,18016 Granada, Spain.

Table of contents

Figure S1. ^{1}H NMR (300 MHz, CD_3OD) spectrum of compound **1**

Figure S2.^{13}C NMR (75 MHz, CD_3OD) spectrum of compound **1**

Figure S3.HSQC (300 MHz, CD_3OD) spectrum of compound **1**

Figure S4.HMBC (300 MHz, CD_3OD) spectrum of compound **1**

Figure S5. ^{1}H-^{1}H COSY (300 MHz, CD_3OD) spectrum of compound **1**

Figure S6. ^{1}H-^{1}H ROESY (500 MHz, CD_3OD) spectrum of compound **1**

Figure S7. HRESIMS of compound **1**

Figure S8. IR spectrum of compound **1**

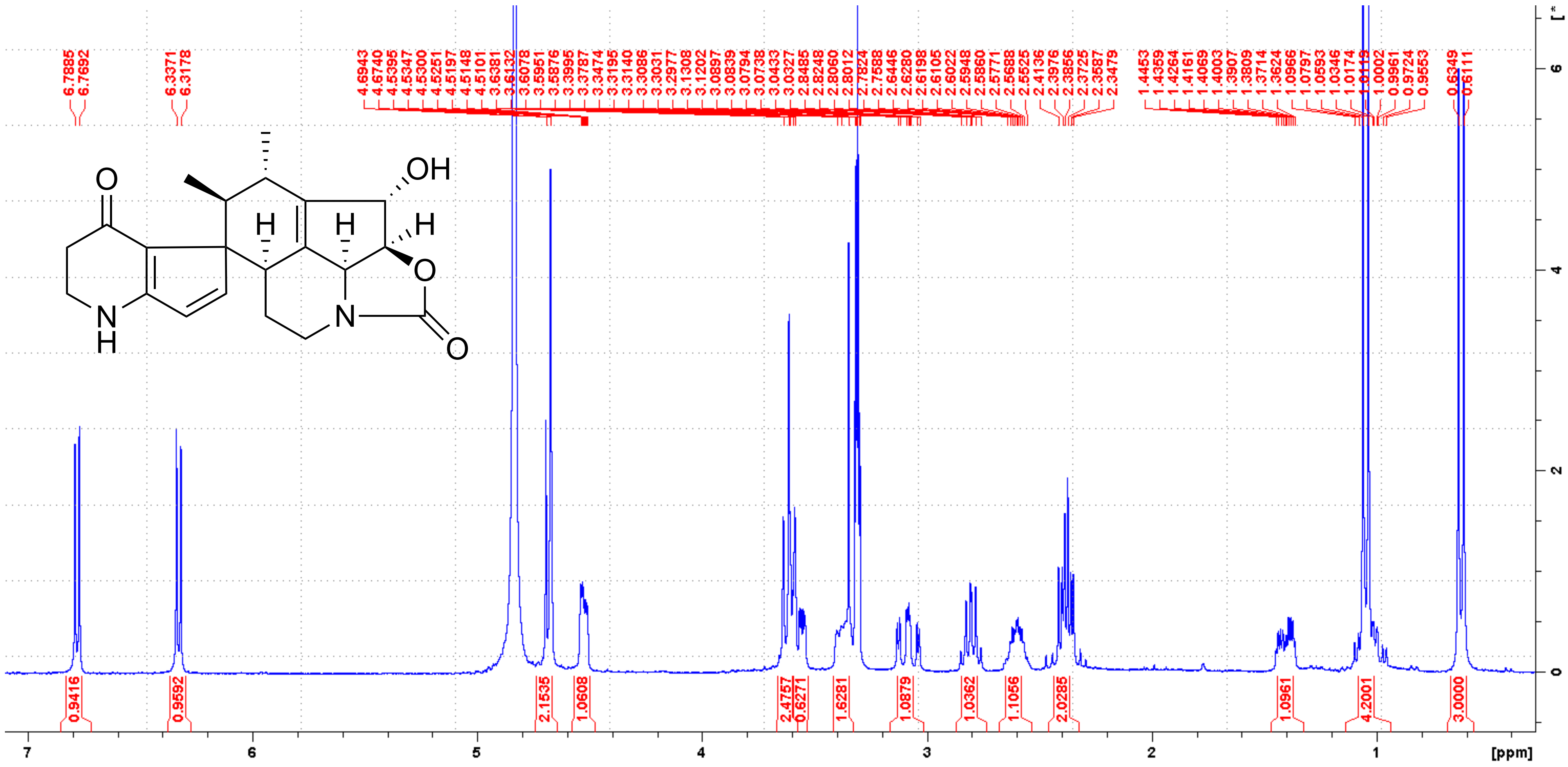

Figure S1. 1H NMR (300 MHz, CD_3OD) spectrum of compound **1**

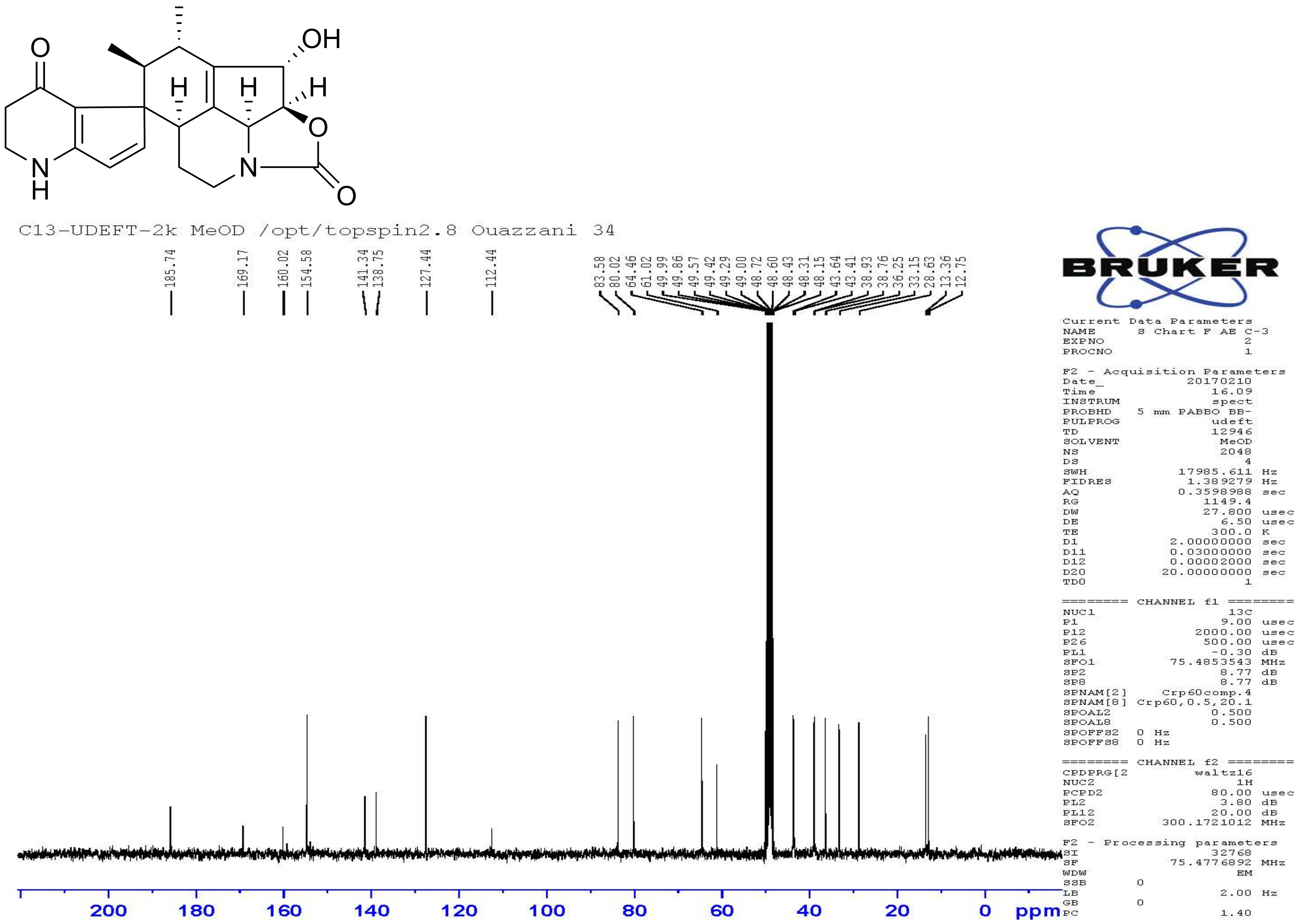

Figure S1. 13C NMR (300 MHz, CD_3OD) spectrum of compound **1**

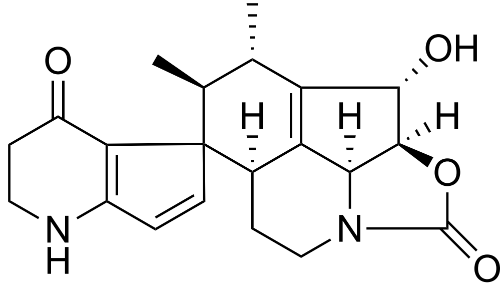

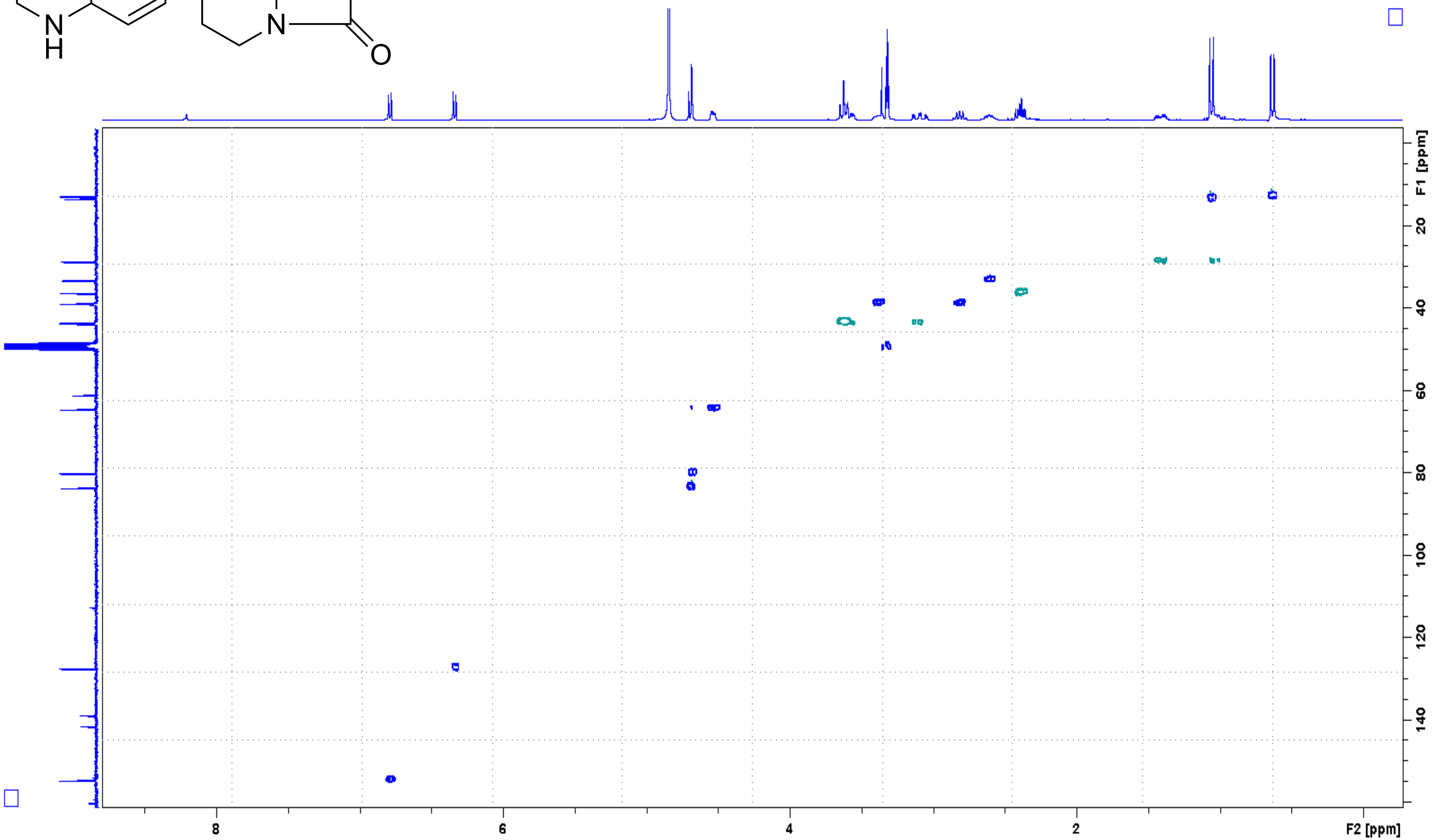

Figure S3. HSQC (300 MHz, CD_3OD) spectrum of compound **1**

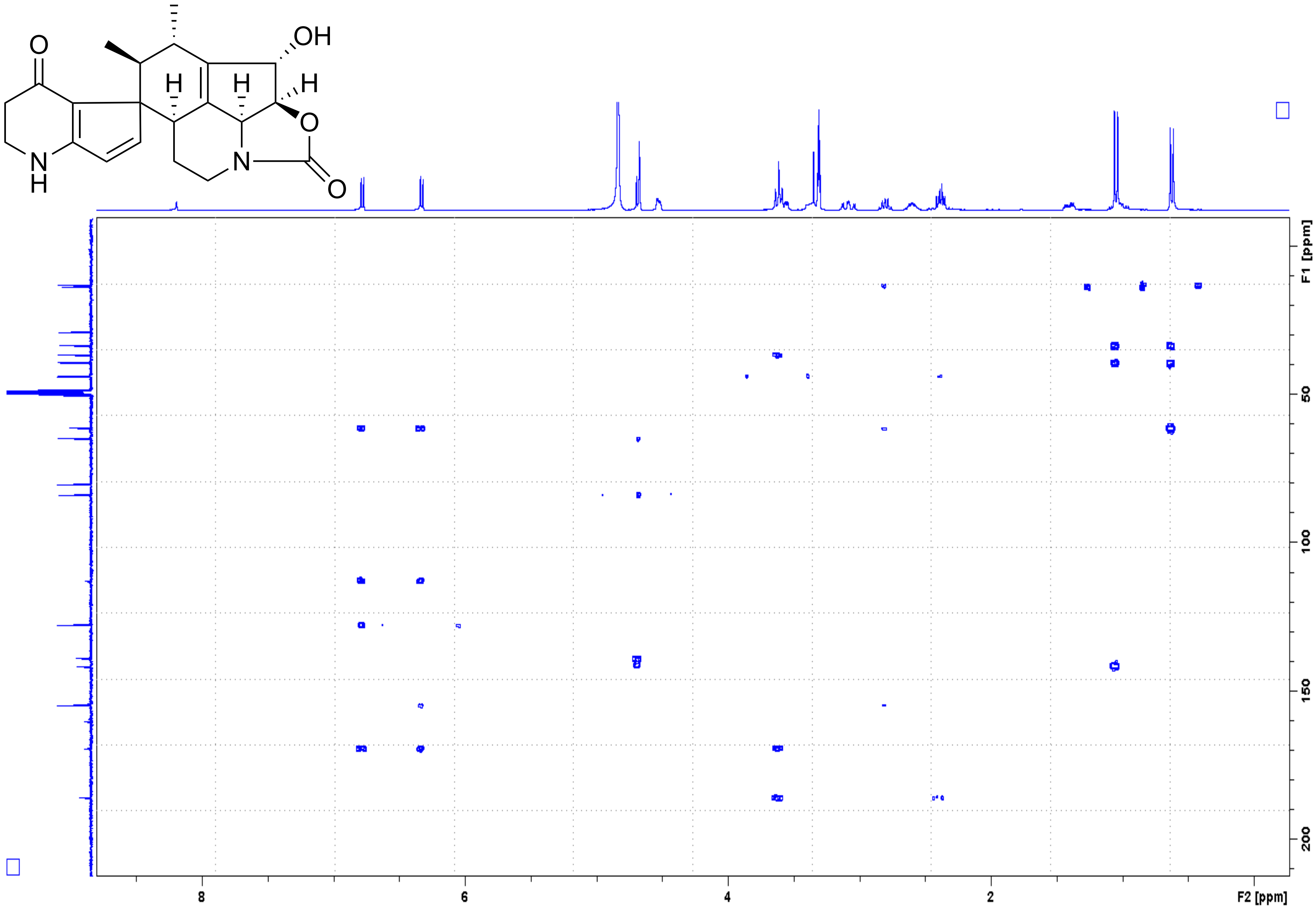

Figure S4. HMBC (300 MHz, CD_3OD) spectrum of compound **1**

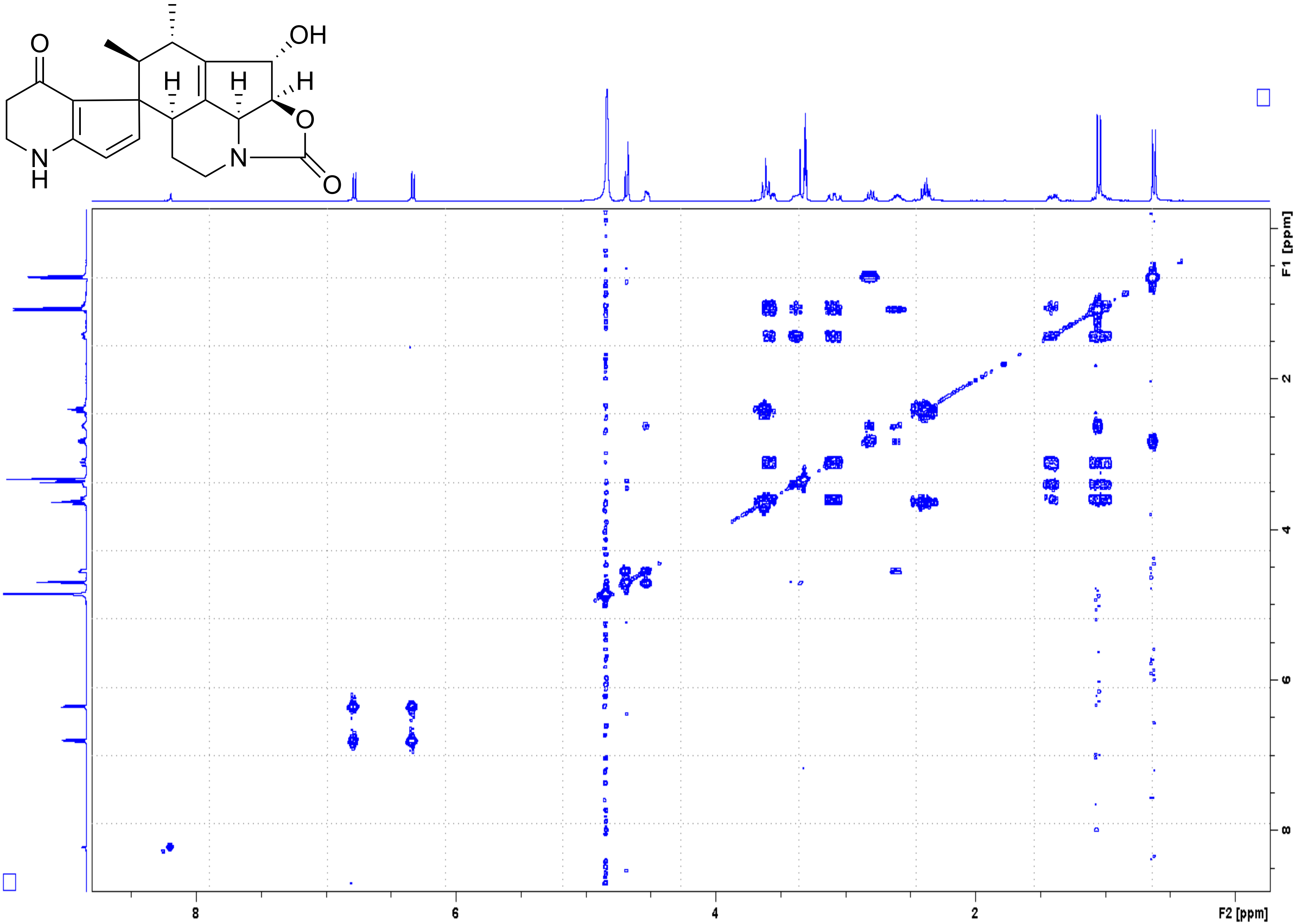

Figure S5. COSY (300 MHz, CD_3OD) spectrum of compound **1**

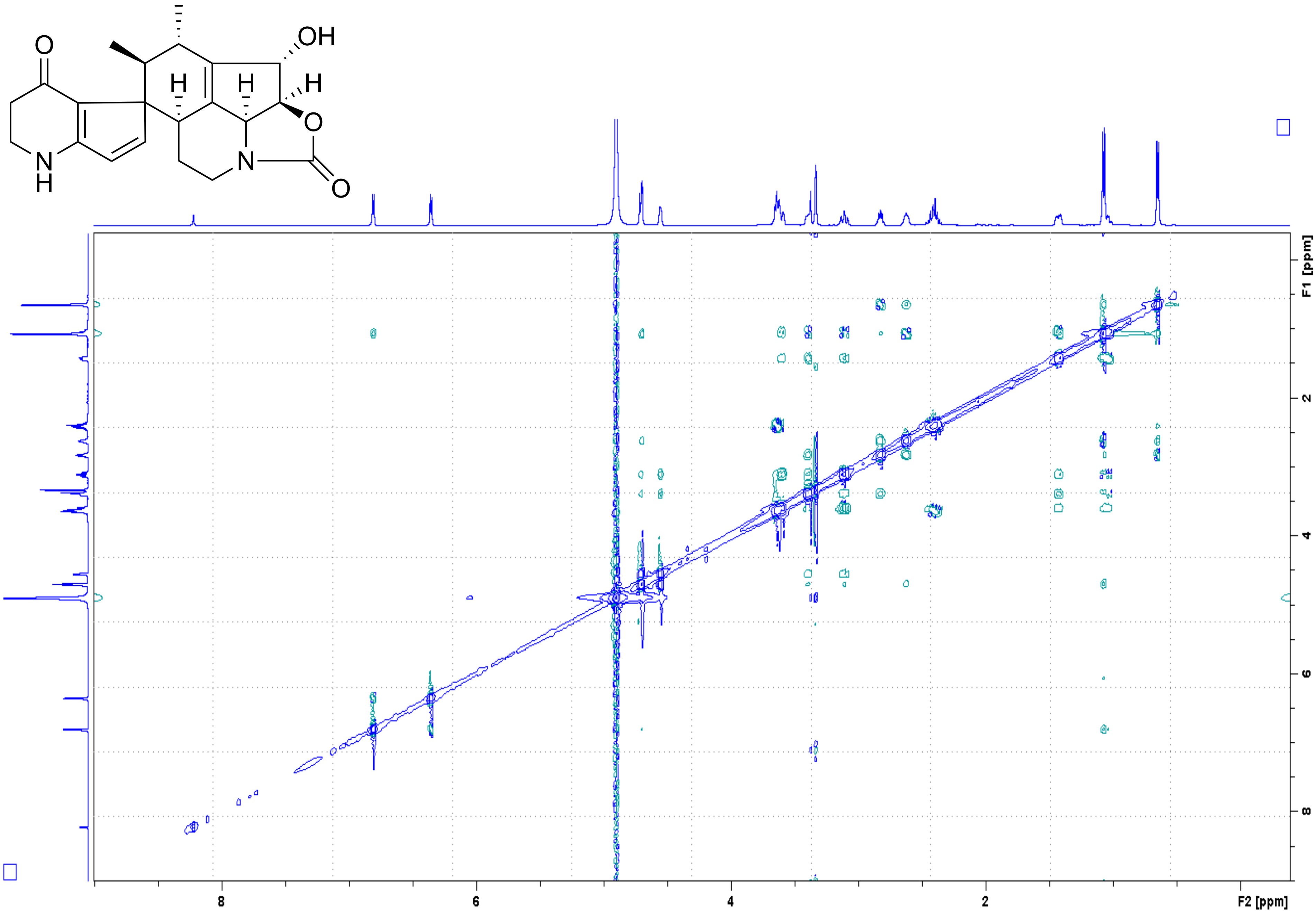

Figure S6. ROESY (500 MHz, CD_3OD) spectrum of compound **1**

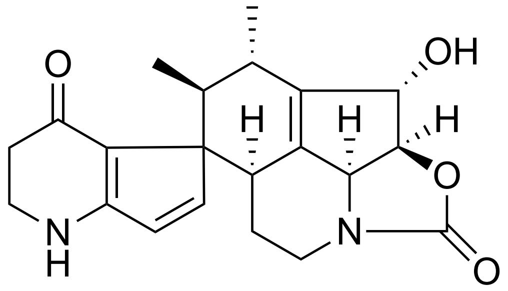

Elemental Composition Report

Single Mass Analysis

Tolerance = 10.0 PPM / DBE: min = -1.5, max = 100.0
Element prediction: Off
Number of isotope peaks used for i-FIT = 9

Monoisotopic Mass, Even Electron Ions
917 formula(e) evaluated with 11 results within limits (all results (up to 1000) for each mass)
Elements Used:
C: 0-100 H: 0-120 N: 0-10 O: 0-10 Na: 0-1

08-Feb-2017 17:38:49 LCT Premier XE KE483 OUAZZANI_eskandar4-3 27 (0.698)
1: TOF MS ES+

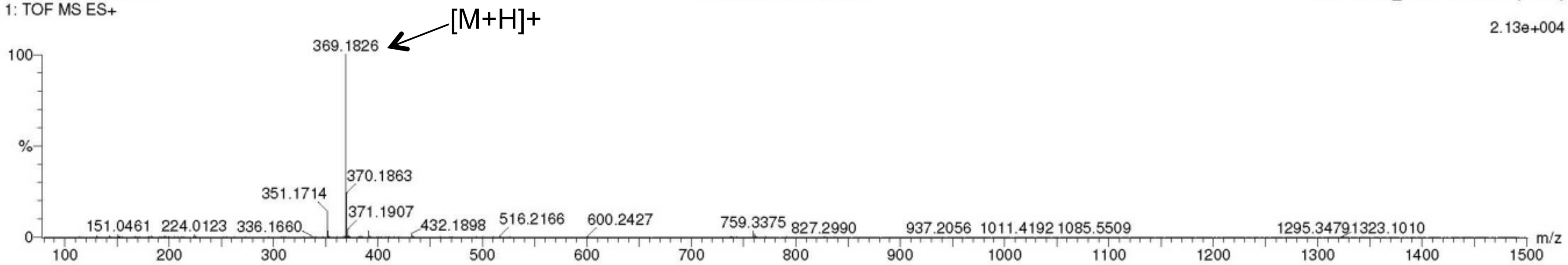

Mass	Calc. Mass	mDa	PPM	DBE	i-FIT	i-FIT (Norm)	Formula
Minimum:				-1.5			
Maximum:		5.0	10.0	100.0			
369.1826	369.1828	-0.2	-0.5	15.5	417.6	3.5	C22 H21 N6
	369.1822	0.4	1.1	-0.5	426.9	12.8	C8 H26 N8 O7 Na
	369.1831	-0.5	-1.4	11.5	416.6	2.5	C24 H26 O2 Na
	369.1814	1.2	3.3	10.5	414.2	0.2	C21 H25 N2 O4
	369.1846	-2.0	-5.4	2.5	424.9	10.9	C10 H25 N8 O7
	369.1806	2.0	5.4	-1.5	429.0	14.9	C5 H25 N10 O9
	369.1804	2.2	6.0	12.5	418.2	4.2	C20 H22 N6 Na
	369.1849	-2.3	-6.2	-1.5	424.3	10.3	C12 H30 N2 O9 Na
	369.1855	-2.9	-7.9	14.5	419.0	5.0	C26 H25 O2
	369.1862	-3.6	-9.8	3.5	423.0	9.0	C13 H26 N6 O5 Na
	369.1790	3.6	9.8	7.5	416.9	2.8	C19 H26 N2 O4 Na

Figure S7. HRESIMS of compound **1**

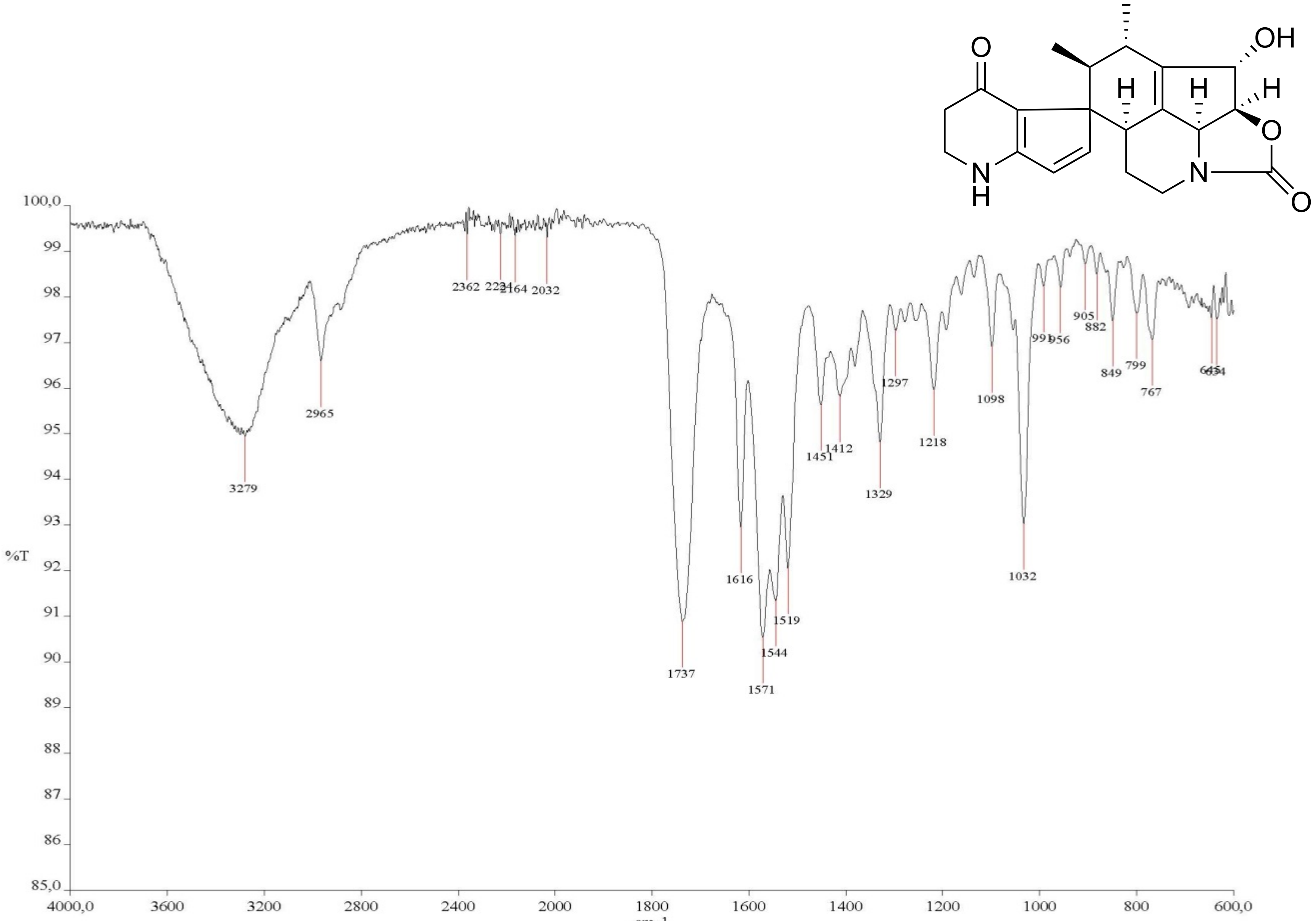

Figure S8. IR spectrum of compound **1**
